# Sustainable Foliar Applications to Improve Grapevine Responses to Drought, High Temperatures, and Salinity: Impacts on Physiology, Yields, and Berry Quality

**DOI:** 10.3390/plants14142157

**Published:** 2025-07-13

**Authors:** Despoina G. Petoumenou, Vasiliki Liava

**Affiliations:** Laboratory of Viticulture, Department of Agriculture, Crop Production and Rural Environment, University of Thessaly, 38446 Volos, Greece

**Keywords:** biostimulants, heat stress, seaweed, yeast, protein hydrolysates, kaolin, zeolite, abscisic acid, putrescine, anthocyanins

## Abstract

Environmental challenges such as drought, high temperatures, and salinity compromise grapevine physiology, reduce productivity, and negatively affect grape and wine quality. In recent years, foliar applications of biostimulants, antitranspirants, and phytohormones have emerged as promising strategies to enhance stress tolerance in grapevines. This review focuses on the main effects of salinity, drought, and high temperatures and the combined impact of drought and high temperatures on grapevines and examines how foliar applications influence grapevine responses under these specific stress conditions. Synthesizing the recent findings from the last ten years (160 articles), it provides direct insights into the potential of these compounds to alleviate each type of stress, highlighting their effects on grapevine physiology, yield components, and secondary metabolites in berries. While their mechanism of action is not entirely clear and their efficacy can vary depending on the type of compound used and the grapevine variety, most studies report a beneficial effect or no effect on grapevines under abiotic stresses (either single or combined). Future research is necessary to optimize the concentrations of these compounds and determine the appropriate number and timing of applications, particularly under open-field experiments. Additionally, studies should assess the effect of foliar applications under multiple abiotic stress conditions. In conclusion, integrating foliar applications into vineyard management represents a sustainable technique to mitigate abiotic stresses associated with climate change, such as salinity, water deficit, and heat stress, while preserving or enhancing the quality of grapes and wines.

## 1. Introduction

Grapevine (*Vitis vinifera* L.) belongs to the *Vitaceae* family and is one of the most economically important fruit crops worldwide [1]. In 2024, the global area covered by vineyards is approximately 7.1 million hectares, and global wine production, excluding juices and musts, is estimated to be 225.8 mhl (OIV) [2]. Grapevines are perennial woody plants well suited to Mediterranean climates but can also adapt to temperate and semi-dry regions. However, despite their adaptability, the productivity and quality of grapes and wines are affected by several climate factors. Therefore, the challenges posed by extreme weather conditions due to climate change represent a significant threat to grapevine growth [3].

Climate change has significantly affected weather patterns, resulting in profound effects on the agricultural sector [4]. The frequent occurrence of extreme weather events, such as high temperatures, increased solar radiation, and drought conditions, poses a serious threat to crop productivity [5,6]. In Mediterranean viticultural areas, projections suggest that worsening droughts and heatwaves will likely increase the stress on grapevines, limiting their productivity and berry quality [7].

Grapevines exposed to multiple summer stresses demonstrate a decline in CO_2_ assimilation and a decrease in the allocation of dry matter to sink organs [8]. Prolonged exposure to these limiting factors can lead to the dysfunction of photosystem II, resulting in yellowing leaves, followed by necrosis and abscissions [8]. These conditions can significantly restrict the growth and productivity of grapevines, impacting the quality of the grapes and the wines produced [9,10,11,12,13,14]. In addition, the unpredictability of intense rainfall may lead to waterlogging, creating further challenges for grapevine cultivation [15].

In response to the growing challenges of climate change, there has been an increasing focus on sustainable practices that utilize cost-effective mitigation strategies, minimizing the use of chemical inputs [16,17,18]. These practices aim to maintain or improve grapevine productivity; enhance the quality of grapes and wines; and increase tolerance to abiotic stressors [19]. Examples include the use of more tolerant rootstocks; the selection of resilient cultivars and resistant interspecific hybrids, as well as improvements in canopy management and irrigation systems; and the application of specialized protective compounds [6,20].

Among the most promising strategies are biostimulants, which enhance grapevine physiology and metabolism, thereby alleviating abiotic and biotic stresses [21]. Additionally, phytohormones, elicitors, osmoprotectants, and trace elements play a crucial role in mitigating the effects of stress on grapevines [3]. These compounds protect and strengthen grapevines, contributing to environmental sustainability in viticulture [22].

Therefore, the aim of this review is to provide a comprehensive evaluation of the recent research on the effectiveness of foliar applications in alleviating the negative impacts of abiotic stress, such as drought, high temperatures, and salinity, on viticulture. It briefly presents the physiological and biochemical responses of grapevines to these stress conditions. The review also highlights the implications of foliar applications for grapevines and their role in enhancing crop resilience and maintaining the quality of grapes and wines under challenging environmental conditions. This review offers original insights into how foliar applications affect grapevine physiology, growth, productivity, and berry quality under specific stress conditions. It provides valuable knowledge on the effectiveness of various compounds in mitigating the negative effects of each type of abiotic stress. In conclusion, this study contributes to the advancement of sustainable viticulture by demonstrating how foliar applications of these compounds can mitigate the effects of these abiotic stresses, thereby reducing the need for excessive irrigation and chemical applications. These environmentally friendly approaches support resource-efficient grape production and align with global sustainability targets, particularly those outlined in the United Nations Sustainable Development Goals (UN SDGs).

## 2. Abiotic Stresses

### 2.1. Salinity and Alkalinity Stress

Soil salinization primarily occurs due to several factors, including high rates of soil water evaporation, inadequate rainfall, poor-quality irrigation water, and the indiscriminate application of fertilizers. High soil salinity poses significant challenges for grapevines, affecting water uptake and nutrient availability. This leads to disrupted ion homeostasis, increased concentrations of toxic ions, and degradation of the soil structure [23,24]. Under salinity stress, grapevines exhibit a decrease in the relative water content in their leaves and reductions in key nutrient concentrations (N, P, K), alongside a declining K^+^/Na^+^ ratio. As salinity levels increase, the sodium percentage rises, which can lead to membrane dysfunction, associated with high membrane permeability [25]. In response, grapevines accumulate osmoprotectants such as glycine betaine and trehalose. This accumulation helps to maintain high K^+^ levels and consequently lower K^+^/Na^+^ ratios. These adaptations are crucial for preserving photosynthetic efficiency and maintaining cell water status under saline conditions [26].

Salinity stress negatively impacts grapevine physiology, leading to reductions in photosynthesis rates, pigment concentrations, chlorophyll fluorescence, and growth [24,25,27,28]. These effects are primarily due to the excessive production of reactive oxygen species (ROS), such as superoxide anions (O_2_^−^) and hydrogen peroxide (H_2_O_2_), which disrupt photosynthesis and cause oxidative damage to the cellular components [26]. Additionally, salinity stress increases the levels of malondialdehyde (MDA), proline, and soluble sugars, the total phenolic content, and antioxidant enzyme activity in grapevine leaves [25,28,29].

Salinity has a notable impact on berry quality, as it reduces organic acid levels while increasing sugars, total phenols, and anthocyanin accumulation [30]. Additionally, proanthocyanidins also show an increase under high-salinity conditions [31]. In summary, salt stress presents significant challenges for grapevines, leading to various biochemical and metabolic changes due to oxidative stress and a reduced osmotic potential, which disrupt metabolism, growth, performance, and overall productivity [26,28].

Additionally, the presence of alkalinity can exacerbate the effects of salinity, further hindering plant growth. Under these stressful conditions, the Na^+^ content increases, disrupting the balance of Na^+^/K^+^, while the expression of VvNHXP (NA^+^/H^+^ antiporter) and VvHKT2 (potassium transporter) is downregulated. Osmotic stress, ionic toxicity, and high-pH stress result in water loss in plant cells and affect their normal metabolic activity [32]. Thus, alkaline stress, which can be even more damaging than salinity, reduces chlorophyll content and photosynthetic capacity, severely affecting grapevine vitality [33]. Although grapevines are known for their ability to tolerate salt and alkali and can utilize saline–alkali soil, exposure to severe saline–alkali environments can negatively affect the yield and quality of grapes. Therefore, it is crucial to enhance grapevines’ tolerance to these stresses [32]. Transcriptome analyses indicate that grapevines respond to alkalinity by activating ‘Plant hormone signal transduction (ko04075)’ and ‘MAPK signaling pathway-plant (ko04016)’ [33].

### 2.2. Water Stress

Drought is recognized as a significant environmental stress, especially in arid and semi-arid regions [34]. Grapevine is considered a drought-resistant species due to its adaptation to semi-arid conditions [35]. However, there are considerable differences among cultivars in their drought tolerance, highlighting the need for further investigation [36,37,38].

The challenge of the water deficit due to climate change highlights the importance of developing effective irrigation systems to mitigate drought-induced yield losses in grapevines [39]. Deficit irrigation can increase water use efficiency by ensuring that crops receive the optimal amount of water at the appropriate time [40]. Moderate water stress has been shown to improve quality parameters such as the total anthocyanins and phenolic content in berries, subsequently contributing to wine composition [39]. However, under ongoing climate change, grapevines are likely to face prolonged water deficits, negatively affecting yield and fruit quality [35].

Drought stress significantly impairs grapevine growth by affecting several pathways, including plant hormone signal transduction, proline metabolism, the biosynthesis of secondary metabolites, and the suppression of photosynthesis [37,41]. Under water deficit conditions, impairments in the photosynthetic process can lead to physiological and biochemical changes, resulting in the excessive generation of reactive oxygen species (ROS) [38]. To mitigate oxidative damage, grapevine enhances the activity of antioxidant enzymes such as guaiacol peroxidase (GPX), superoxide dismutase (SOD), ascorbate peroxidase (APX), and catalase (CAT). Also, there is a notable increase in the accumulation of abscisic acid (ABA), proline, the level of hydrogen peroxide (H_2_O_2_), and malondialdehyde (MDA) [14,42,43].

Among the endogenous hormones involved in grapevine responses to drought conditions, abscisic acid (ABA) is recognized as the most significant [34]. Water stress signals are transmitted to the leaf tissues through ABA produced by the roots, leading to changes in stomatal pore aperture that reduce transpiration in grapevines [44]. Consequently, water stress conditions are associated with a decline in chlorophyll and carotenoid content [34,42]. Grapevine vegetative growth is also hindered since the development of the primary and lateral shoots is negatively affected in grapevines experiencing water stress [45]. This results in a decreased leaf area and an increased canopy temperature [46]. Water deficit can reduce berry weight and size and overall fruit yield [35,42,47,48], with the impact varying based on the severity of stress and the timing of its occurrence [49].

The effects of water stress on quality parameters in grapes can vary depending on cultivar. Several studies indicate that water deficit can lead to increased levels of titratable acidity (TA) and total soluble solid (TSS) contents in grapes [35,42,50], while other studies have reported TA and TSSs that either decreased or remained stable [39,51]. Similarly, Chrysargyris et al. [52] reported that water stress resulted in increased and decreased TA across different grape varieties. It is important to note that the severity and timing of deficit irrigation significantly influence grape quality. Specifically, mild water stress applied to grapevines approximately 20 days after anthesis can increase monosaccharide concentrations, particularly glucose, during the veraison stage [46].

Water stress also influences the accumulation of secondary metabolites, particularly the phenolic content, in berries. Griesser et al. [53] and Sikuten et al. [54] noted an increase in polyphenols under prolonged drought stress. This increase is attributed to a reduced berry size, which enhances the ratio of the berry’s skin (where many phenolics accumulate) to its flesh [47,55]. Deficit irrigation may enhance anthocyanin biosynthesis, although the outcomes depend strongly on the severity of the water deficit [56]. In contrast, Chacón-Vozmediano et al. [57] found no significant effect of water stress on anthocyanins, catechins, tannins, or total polyphenols in berry skin. Furthermore, severe water deficits may negatively impact the accumulation of phenolics, including anthocyanins [16]. In grapevines, the influence of water stress on the secondary metabolism is highly genotype- and timing-specific. Further research is needed to identify the thresholds for positive versus negative effects across cultivars [58].

### 2.3. Heat Stress

Solar radiation and temperature are critical factors in the metabolic processes of grapevines [59]. Climate change has increased the frequency, duration, and intensity of heatwaves in many key wine-producing regions around the world [60]. Heat stress causes the oxidation of nucleic acids, lipids, and proteins, leading to cellular, physiological, molecular, and biochemical disruptions in the grapevine [61]. As a result, this stress has become one of the most significant threats to viticulture, negatively affecting grape quality and production [62,63].

High temperatures inhibit nutrient metabolism, hinder growth, and create imbalances in energy metabolism [63]. Additionally, heat stress can compromise the structural integrity of grapevine leaves, impairing photosynthesis, yield, and fruit quality [8,62,64]. Elevated temperatures accelerate grape ripening [65], leading to earlier harvests—especially when heat occurs during the ripening phase [66]. The influence of temperature is more pronounced at the onset of veraison compared with that in the period from mid-veraison to maturity [67]. As global temperatures rise, key developmental stages in grapevines may occur earlier than usual, disrupting the balance of sugars and acids, which can impact their quality [68]. Specifically, under heat stress, grape berries accumulate sugars while losing acidity at an accelerated rate, resulting in wines that may have higher alcohol contents and lower acidity [3,69]. Additionally, increased temperatures are correlated with a decrease in anthocyanin accumulation in the skin of grape berries, a key factor influencing the quality of red wine [70]. Anthocyanin biosynthesis is regulated by the abscisic acid (ABA)-to-gibberellin (GA) ratio, which is disrupted by high temperatures [71] and inhibited at temperatures above 30–33 °C [72]. Additionally, grape berries have become more susceptible to sunburn damage [12], and prolonged heat exposure can reduce the temperature threshold required to induce damage [73].

### 2.4. A Combination of Water Deficit and Heat Stress

Viticulture is increasingly facing challenges from high temperatures, heatwaves, and drought, which can significantly influence grapevines’ growth, maturity, and overall quality. Research is now focused not only on the effects of individual stressors but also on the interactions between multiple stresses that may occur sequentially during the same growing period [74]. Recent findings suggest that heat stress, whether experienced alone or in combination with water stress, has a more significant impact on the organic acid content, pH, and TA of grape berries compared to that of water stress alone. This is in contrast to previous studies that indicated that the leaves respond more to water stress, highlighting that grape berries have a unique, organ-specific response to environmental stresses [75]. Therefore, understanding the interaction between different stressors is crucial for developing more effective adaptation strategies in viticulture.

## 3. Biostimulants, Phytohormones, and Reflective Antitranspirants

In viticulture, the foliar application of biostimulants, phytohormones, and reflective antitranspirants has emerged as a sustainable strategy to enhance grapevines’ resilience to abiotic stresses and improve overall plant growth, productivity, and grape and wine quality. According to du Jardin [76], plant biostimulants are defined as substances or microorganisms applied to plants that improve their nutrition efficiency, increase their tolerance to abiotic stress, and enhance crop quality traits, regardless of nutrient content. Foliar applications allow for reduced quantities of these products and promote rapid and direct absorption through the leaves [77]. Their effectiveness is linked to various physiological and molecular mechanisms that influence biochemical activity in plants [78,79], although the exact mechanisms of action are still only partially understood [80].

The main categories of foliar biostimulants include humic and fulvic acids, protein hydrolysates, seaweed extracts, and chitosan [76]. Humic substances, which consist of humic acid and fulvic acid, are formed through the microbial decomposition of plant and animal matter [81]. These substances have been shown to positively affect grapevine growth and improve berry quality parameters such as TSS and TA [77]. Protein hydrolysates, derived from byproducts of the vegetable and food industries [82], are rich in growth regulators such as cytokinins, auxins, and other important molecules, including betaines, polyamines, peptides, and oligosaccharides. These components contribute to enhanced plant vigor and stress tolerance [83]. Seaweed and microalgae, which are marine-derived substances, enhance quality and resistance to abiotic stress through various mechanisms [84]. They are among the most studied compounds in agriculture [85] since they are rich in growth-promoting compounds, amino acids [86], macro- and micronutrients, and organic compounds [87]. Their effectiveness is attributed to the interactions among their components, rather than the actions of individual components [88]. Seaweed has been shown to enhance antioxidant defense by effectively lowering reactive oxygen species (ROS) levels through stimulation of the ascorbate (AsA)–glutathione (GSH) cycle [89]. Metabolomic analyses reveal that seaweed increases the accumulation of stress-protective primary and secondary metabolites such as GABA, proline, maltose, ascorbic acid, and quercetin under abiotic stress. Combined transcriptome and metabolome analyses indicate that seaweed preserves photosynthetic activity and promotes the accumulation of protective metabolites and amino acids, thereby supporting plant survival and growth [90]. Chitosan, a natural polymer of b-1,4-D-glucosamine, is a polysaccharide obtained from the partial deacetylation of chitin (from 15% to 90%). Chitin is a natural structural compound found in the cell walls of several fungi, as well as in the exoskeleton of crustaceans and the cuticles of insects [91,92,93].

In the quest for more sustainable viticulture, Monteiro et al. [21] emphasized the importance of discovering new biostimulant compounds. The definition of biostimulants varies depending on the regulatory context. Many biostimulants are plant extracts [94], but they can also include other products, such as yeast extracts. Yeast and inactivated yeast act as elicitors under unfavorable climatic conditions [69,95]. Moreover, osmoprotectant compounds, including amino acids, sugars, and polyols, are gaining attention as important biostimulants [96]. Amino acids, typically derived from plant and animal sources, consist of a mixture of amino acids, peptides, polypeptides, and denatured proteins, all contributing to improved stress tolerance [42]. Polyamines, like putrescine and spermidine, characterized by the presence of two or more primary amino groups, have also shown the potential to enhance stress tolerance [97]. These compounds regulate antioxidant defense mechanisms, preserve cell integrity, and improve key metabolic functions across different plant species [98]. They perform diverse functions in biological processes, including the regulation of gene expression, translation, cell proliferation, and the modulation of cell signaling [99].

In addition to biostimulants, several phytohormones such as abscisic acid (ABA), gibberellic acid (GA3), jasmonic acid (JA), and salicylic acid (SA) have been shown to effectively mitigate the negative effects of summer stress in grapevines [3]. Methyl jasmonate, a volatile compound derived from *Jasminum grandiflorum* [100], and brassinosteroids, which are naturally occurring polyhydroxylated steroidal phytohormones [43], have also been studied for their role in enhancing plant responses to environmental stress. Phytohormones play a crucial role in enhancing grapevine survival under stress by participating in complex regulatory mechanisms. They modulate various biological processes and influence the expression of specific genes through upregulation or downregulation [101]. For instance, JA promotes the upregulation of genes involved in proline production, leading to increased osmolyte production. Similarly, SA increases the production of proline and glycine betaine, while ABA plays a significant role in the stomatal closure process and the regulation of gene expression [101]. Moreover, their exogenous application is important as they can upregulate the transcript levels of mitogen-activated protein kinases (MAPKs) [33,102]. MAPKs are signaling molecules that mediate the intracellular transmission of extracellular signals, resulting in the induction of appropriate cellular responses [103]. In plants, MAPK cascades are vital for regulating responses to abiotic stress [104].

Trace elements such as selenium and silicon have proven beneficial in alleviating the negative effects of abiotic stresses [3]. One promising method for managing radiation is the foliar application of reflective antitranspirants, such as kaolin, a chemically inert white clay mineral. Kaolin increases the reflection of ultraviolet (UV) and infrared (IR) radiation, which helps lower leaf temperature and reduce transpiration [81,105], which can be particularly beneficial during periods of heat and drought.

In conclusion, the increasing impact of climate change has prompted scientists to develop sustainable viticulture strategies, including foliar applications of compounds that enhance plant resilience. Future research should aim to test these compounds across various grapevine cultivars while optimizing the application timing, dosage, frequency, and phenological stages, as well as assessing their effectiveness under specific environmental conditions [21,106]. The following sections provide a comprehensive overview of the major effects of foliar applications on grapevine performance and quality in response to salinity, drought, heat stress, and combined water and heat stress. The final section offers a brief summary of the effects of these compounds on quality parameters and the accumulation of secondary metabolites.

### 3.1. The Effect of Foliar Applications Under Salinity and Alkalinity Stress

Recent studies have shown that various foliar treatments, including silicon, abscisic acid, putrescine, spermidine, brassinolide, and seaweed extracts, can effectively alleviate the adverse effects of salinity and alkalinity stress on grapevines (Table 1). These compounds significantly improve the water status of grapevines, maintain higher photosynthetic rates, and preserve chlorophyll levels under salinity conditions. Additionally, they enhance the antioxidant defense system, thereby reducing oxidative damage. A key mechanism behind their protective effects is the regulation of ionic balance. Most of these treatments decrease Na^+^ accumulation while promoting K^+^ uptake, thereby improving the K^+^/Na^+^ ratio.

In grapevines, silicon mitigates the negative effects of salinity by reducing the uptake of Na^+^ and Cl^−^, with the reduction in Cl^−^ being particularly important for improving plant performance under salinity stress. The enhanced photosynthesis capacity under salinity may be attributed to the regulation of the ROS levels in plant cells and the reduction in Cl^−^. Additionally, silicon activates antioxidant enzymes and regulates the accumulation of H_2_O_2_, which prevents lipid peroxidation induced by salinity [24]. It also protects the chloroplasts and, through the transformation of sugars into starch, can help plants avoid the metabolic disruptions caused by inhibition due to excess amounts of sucrose in the cytoplasm [27]. The application of ABA enhances grapevines’ tolerance to salinity by increasing the activities of antioxidant enzymes, as well as the chlorophyll content, phenols, flavonoids, proline, and total sugar content [29]. Similarly, the use of brassinolide in grapevines improves the chlorophyll and proline contents and the activities of key antioxidant enzymes to scavenge ROS; enhances cell membrane stability and nutrient uptake; maintains water status; and preserves the ultrastructure of the cell organelles and leaf anatomy [25].

Spermidine enhances grapevines’ tolerance to salinity–alkalinity by upregulating the expression of *VvNHXP* and *VvHKT2*, which promotes K+ uptake and Na+ excretion. This compound also improves the photochemical efficiency of PSII, stimulates the synthesis of soluble sugars, and increases the conversion of other amino acids into proline [32]. Seaweed extract (*A. nodosum*) improves the Zn uptake under alkalinity, which may stimulate the synthesis of indole acetic acid (IAA), a plant growth regulator involved in pollen and berry formation, contributing to improved berry characteristics and yield [85]. ABA enhances grapevine tolerance to alkaline stress at both the physiological and transcriptional levels. Transcriptome analysis shows that exogenous ABA activates multiple pathways, including ‘Plant hormone signal transduction (ko04075)’, ‘MAPK signaling pathway plant (ko04016)’, ‘Photosynthesis-antenna proteins (ko00196)’, ‘phenylpropanoid biosynthesis (ko00940)’, ‘Flavonoid biosynthesis (ko00941)’, and ‘Carotenoid biosynthesis (ko00906)’. It also upregulates genes related to chlorophyll metabolism and ion transport in grapevines under alkaline stress [33]. These improvements contribute to enhanced stress resilience in grapevines. Therefore, incorporating these foliar applications into sustainable viticulture strategies can enhance stress resilience and thereby grapevine performance under salinity and alkalinity stress.

### 3.2. The Effect of Foliar Applications Under Water Stress

The reviewed studies demonstrate that the foliar application of biostimulants, phytohormones, antitranspirants, and related compounds significantly enhances the drought tolerance of grapevines through a range of physiological and biochemical mechanisms (Table 2). The positive impact of biostimulants on grapevine yields under drought stress can be attributed to their rich composition, which often includes phytohormones, vitamins, and minerals. These treatments have been shown to improve leaf water status, enhance photosynthetic efficiency, increase the activity of antioxidant enzymes, and promote the accumulation of osmolytes. The increased activity of antioxidant enzymes, such as CAT, SOD, and POD, effectively mitigates the oxidative stress caused by ROS, thus protecting the cellular structures from damage. The accumulation of osmolytes, such as proline, plays a crucial role in maintaining cell turgor and osmotic adjustment under water deficit conditions.

Under severe drought conditions, the application of kaolin preserves the integrity of the photosynthetic apparatus by reducing leaf temperature, which prevents complete stomatal closure in grapevine leaves. Additionally, cooling of the berry’s skin contributes to maintaining berry color [107]. Seaweed extracts, particularly those derived from *A. nodosum*, have been shown to increase carbohydrate concentrations in grapevine leaves. This increase supports a higher photosynthetic rate and helps protect photosystem II [108]. Such treatments also improve plants’ water status by preventing excessive stomatal closure [109]. Protein hydrolysates have been found to upregulate enzymes related to photosynthesis, thereby enhancing vegetative growth and nutrient uptake in grapevines under drought stress [80]. Similarly, the application of melatonin significantly increases the accumulation of osmolytes in the leaves, which aids in osmotic adjustment and maintains membrane integrity. It also reduces reactive oxygen species (ROS) levels and boosts the activities of antioxidant enzymes (SOD, POD, and CAT), along with enhancing the AsA-GSH cycle, thereby mitigating oxidative damage [110]. In grapevines, brassinolide supports the chloroplasts and photosynthetic performance by promoting the accumulation of AsA and GSH, key components in the detoxification of ROS. It upregulates AsA biosynthesis genes, such as *VvGME2*, *VvMIOX2*, and *VvGGP1*, and downregulates the catabolism gene *VvAO* [43]. Putrescine, an osmotically active substance, reduces ABA accumulation, thereby maintaining and regulating homeostasis through its antioxidant and ROS scavenging properties [111]. Strigolactones have also been shown to reduce photosystem damage and regulate both chlorophyll composition and hormonal balance in grapevines under drought stress [112]. The application of exogenous auxin increases the production of osmoregulatory compounds, such as proline and total soluble carbohydrates. This process decreases osmotic potential and enhances the relative water content of the leaves. Auxin also promotes chlorophyll synthesis and photosynthetic efficiency, which contribute to better growth characteristics [41]. Finally, silicon nanoparticles enhance drought tolerance through several mechanisms, including the upregulation of antioxidant enzymes (SOD, CAT, APX); increased synthesis of phenolic compounds, proline, and proteins; and overall improved oxidative stress management [14]. However, it is important to note that most of these findings are based on pot experiments, and open-field experiments should be conducted to validate the results.

**Table 2:** Effect of foliar applications on grapevines under water stress.

Country	Cultivar	Irrigation	Foliar Application	Results	Ref.
Italy (pot experiment)	Sangiovese	Well-watered, water deficit followed by re-watering	Kaolin (3%)	Water relations: ↑ WUE Photosynthesis and gas exchange: ↓ leaf temperature, protected leaf function, protected clusters from overheating and sunburn Quantity traits: − yield and berry weight Quality traits: − TSS, pH, TA, malic acid, and tartaric acid Secondary metabolites: ↑ anthocyanins and phenolics	[107]
Italy (open-field experiment)	Merlot	Well-watered, water deficit (90% and 35% of estimated crop water demand (ETc))	Kaolin-based reflective film (berries at pea size for 3 consecutive weeks at a dosage of 60 g/L in 950 L/ha)	Quantity traits: ↓ number of berries per cluster, ↑ berry fresh weight Quality traits: − TSS and TA Secondary metabolites: ↑ anthocyanins	[113]
USA (pot experiment)	Pinot noir	Well-watered, water deficit followed by re-water	Foliar and soil application of *Ascophyllum nodosum* extract	Water relations: ↑ leaf water potential Photosynthesis and gas exchange: ↑ leaf gas exchange, stomatal conductance, photosynthesis, WUE Osmotic adjustment: ↑ sugar content Recovery after rewatering: Faster physiological recovery Foliar application: Best for rapid stress mitigation, especially in leaves and in photosynthesis Soil application: Better for long-term support, especially for roots and sustained drought adaptation	[108]
Iran (open-field experiment)	Yaghouti	Well-watered, water deficit (irrigation after 60 and 100 mm of evaporation from early April to mid-October)	Amino acid, fulvic acid, and seaweed extract (0.5% AA, FA, and SE applied to millet-sized berries and 2 weeks later) and humic acid (20 g HA /vine at bud swelling and to millet-sized berries)	Chlorophyll and pigments: ↑ chlorophyll content Oxidative stress and antioxidants: ↓ H_2_O_2_ and MDA, ↑ activity of GPX and CAT Osmotic adjustment: ↑ proline, soluble carbohydrates, and proteins Mineral contents: ↑ N, P, K, Fe, and Zn in leaves Quality traits: ↑ weight of 20 berries and yield Quality traits: ↓ TSS and TA Effectiveness: SE was the most effective treatment	[42]
Italy (pot experiment)	Pinot noir	Well-watered, water deficit post-veraison (90% and 40% of the maximum water availability)	*Ascophyllum nodosum* extract (3 g/L, 20 and 14 days before the expected harvest)	Water relations: ↑ leaf water potential Photosynthesis and gas exchange: ↑ photosynthetic rate and stomatal conductance Secondary metabolites: ↑ total phenolics, flavonoids, anthocyanins Upregulation of phenylpropanoid pathway genes related to stress defense	[109]
Italy (pot experiment)	Montepulciano	Full irrigation followed by progressive water stress and then full irrigation restored	Vegetable-derived protein hydrolysates ‘Trainer’ (PH1) and ‘Stimtide’ (PH2)	Quantity traits: − yield Quality traits: ↓ TSS and pH, ↑ TA (only PH2) Secondary metabolism: − total anthocyanins and phenolics	[80]
China (solar greenhouse experiment)	Red earth	Irrigation treatments (360, 300, 240, and 180 mm)	Melatonin (150 µmol L^−1^ at 0, 30, 60, and 90 days after flowering)	Oxidative stress and antioxidants: ↓ MDA, H_2_O_2_, and O_2_^−^ ↑ activity of SOD, CAT, and POD Osmotic adjustment: ↑ proline, soluble sugars, and proteins Hormonal balance: ↓ ABA and SA, which ↑ under drought stress ↑ IAA, GA_3_, and ZT, which ↓under drought stress Quantity traits: ↑ berry weight and diameter Quality traits: − total soluble sugars, glucose, fructose, and sucrose in berries Secondary metabolism: Variable effect on total phenolics and vitamin C ↑ total flavonoids, ↓ tannins Interaction: Effective across varying water regimes, especially under low irrigation	[110]
Turkey (pot experiment)	1103 Paulsen rootstock	Well-watered, water deficit (70–80% and 30–40% of the field capacity)	Putrescine (0, 0.05, 0.1, and 0.2 mM six weeks after planting)	Water relations: ↑ relative water content Photosynthesis and gas exchange: ↑ stomatal conductance and photosynthetic rate Chlorophyll and pigments: ↑ chlorophyll content (SPAD) Cell membrane integrity: ↓ electrolyte leakage, improved membrane stability Morphological traits: ↑ shoot and root lengths, fresh and dry weights of shoots and roots, leaf area, and number of leaves ↓ drought index Optimal concentration of putrescine: 0.1 mM	[111]
China (pot experiment)	Cabernet Sauvignon	Well-watered, water deficit	Methyl jasmonate (100 μM of MeJA)	Water relations: − relative water content Photosynthesis and gas exchange: ↑ photosynthetic rate Chlorophyll and pigments: ↑ chlorophylls a and b and carotenoids Osmotic adjustment: ↑ proline and soluble sugars Oxidative stress and antioxidants: ↑ activity of SOD and CAT Carbon metabolism: ↑ activity of carbon assimilation enzymes Nitrogen metabolism: ↑ nitrate reductase and nitrogen assimilation	[114]
China (pot experiment)	Cabernet Sauvignon	Well-watered, water deficit	Brassinolide 24-Epibrassinolide (0.2 μΜ of EBR)	Water relations: − relative water content ↑ leaf water potential Photosynthesis and gas exchange: ↑ photosynthetic capacity and stomatal conductance Chlorophyll and pigments: ↑ chlorophyll a, total chlorophyll, and carotenoids Oxidative stress and antioxidants: ↓ MDA, H_2_O_2_, and O_2_^−^ ↑ activity of SOD, CAT, POD, and APX Hormonal balance: ↑ ABA, JA, − IAA, GA_3_ Cell membrane integrity: ↓ electrolyte leakage, improved membrane stability	[43]
China (pot experiment)	Cabernet Sauvignon	7% (*w*/*v*) polyethylene glycol to simulate drought conditions	Strigolactone rac-GR24 (1, 3, and 5 μM)	Water relations: ↑ relative water content Photosynthesis and gas exchange: ↓ stomatal opening ↑ transpiration rate, photosynthetic rate, and WUE Chlorophyll and pigments: ↑ chlorophylls a and b Oxidative stress and antioxidants: ↓ ROS and MDA ↓ activity of SOD, POD, CAT, and APX Cell membrane integrity: ↓ electrolyte leakage, improved membrane stability Hormonal balance: ↓ ABA in leaves and roots which ↑ under drought stress, ↑ MeJA in roots and ↓ in leaves − IAA in leaves and roots, which ↓ under drought stress	[112]
Italy (pot experiment)	Pinot Nero	Well-watered, water deficit after veraison (90% and 40% of the maximum water availability)	*Arthrospira platensis* F&M-C256 extract (3 g L^−1^, 10 and 20 days before the expected harvest)	Photosynthesis and gas exchange: − photosynthesis, stomatal conductance, and WUE Quantity traits: ↑ berry weight Quality traits: − sugar content, TA, and pH Secondary metabolites: − total anthocyanins and total phenolics	[115]
Iran (pot experiment)	Rashe (drought-tolerant), Fakhri (drought-sensitive)	Well-watered, water deficit (90%, 50% of field capacity)	Auxin (0, 50, and 200 mg L^−1^ NAA 25, 50, and 75 days after the onset of drought stress	NAA at 50 mg L^−1^ Water relations: ↑ relative water content Photosynthesis and gas exchange: ↑ photosynthesis, stomatal conductance, and intercellular CO_2_ concentration Chlorophyll and pigments: ↑ chlorophyll a and total chlorophylls, − chlorophyll b Oxidative stress and antioxidants: − MDA, H_2_O_2_, and O_2_- ↑ activity of SOD, CAT, POD, and APX Osmotic adjustment: ↑ proline and total soluble carbohydrates Cell membrane stability: − electrolyte leakage Hormonal balance: − ABA, ↑ IAA Cultivar interaction: NAA mitigated the adverse effects of drought stress, especially in the drought-sensitive cultivar	[41]
Turkey (pot experiment)	Crimson Seedless	Well-watered, water deficit (90–100% and 40–50% of field capacity)	Silicon nanoparticles (0, 1, 10, and 100 ppm)	Water relations: ↑ relative water content Photosynthesis and gas exchange: ↑ stomatal conductance and photosynthetic rate Chlorophyll and pigments: ↑ chlorophyll content Osmotic adjustment: ↓ proline which ↑ under drought stress Oxidative stress and antioxidant defense: ↓ ROS and activity of SOD, CAT, and APX, which ↑ under drought stress Growth parameters: ↑ leaf number and area, shoot and root weights (fresh and dry) Secondary metabolites: ↓ total phenolics which ↑ under drought stress Optimal concentration: 10 ppm	[14]

↑: significant increase; ↓: significant decrease; −: no significant effect.

### 3.3. The Effect of Foliar Applications Under Heat Stress

The impact of heat stress on grapevine performance has been studied under controlled conditions, such as in temperature-regulated rooms and greenhouse. In contrast, research on the effects of biostimulants, phytohormones, and antitranspirants on grapevine growth and productivity under high-temperature stress has been conducted in open-field experiments, where meteorological data were recorded (Table 3). Among the treatments evaluated, kaolin and seaweed extracts have significant benefits in mitigating the adverse effects of heat stress.

Biostimulants promote the accumulation of chlorophyll and inhibit its degradation in the leaves, thereby maintaining high photosynthetic efficiency. This effect is particularly beneficial for grapevines’ growth under high-temperature conditions [63]. The application of kaolin has been shown to increase the photosynthetic rate and transpiration, preventing the inhibition of these processes under high temperatures [118]. Due to its reflective properties, kaolin likely reduces leaf temperature and excessive solar radiation, improving physiological performance. In grapevines, kaolin application also positively influences the secondary metabolism at the transcriptional level [17]. Seaweed extracts have been shown to enhance the biosynthesis of anthocyanins and phenolics in grape skins, with the responses varying depending on the ripening stage [5]. An increased content of flavonoids may offer photoprotection to photosystem II, enhancing the grapevines’ performance under heat stress [121]. Additionally, seaweed extracts result in a higher photosynthetic rate and greater stomatal conductance while maintaining transpiration. This may be linked to the increased K levels observed in this treatment since K plays a key role in stomatal regulation and CO_2_ concentrations, both essential for efficient photosynthesis. Additionally, higher yield values may be attributed to improved grapevine nutrition, with significant increases in macro- and micronutrients and better nutritional balance [87]. In conclusion, these compounds have been shown to improve the photosynthetic efficiency of grapevines and promote the accumulation of secondary metabolites, particularly anthocyanins. Since heat stress can inhibit anthocyanin synthesis and impair berry coloration, foliar applications of these compounds can be an effective strategy for preserving grape quality under high-temperature conditions.

### 3.4. The Effect of Foliar Applications Under Water Deficit and Heat Stress

Water stress frequently occurs with high temperatures, making it essential to understand how grapevines respond to these combined stressors to develop effective adaptation and mitigation techniques [123]. Recent studies have demonstrated that foliar applications of various compounds, including kaolin, zeolite, CaCO_3_, seaweed extracts, and yeast derivatives, can effectively reduce leaf and berry temperatures (Table 4). This reduction helps mitigate thermal stress and enhances physiological performance, particularly photosynthetic activity. The application of zeolite has been linked to increased stomatal conductance values and improved photosynthetic rates, suggesting that zeolite may enhance CO_2_ concentrations at the stomatal level in grapevines [124]. Moreover, zeolite provides protection against high temperatures and improves water use efficiency, which is associated with reduced abscisic acid (ABA) accumulation. This reduction may help to minimize the damage caused by heat stress and sunburn injury [6]. Foliar applications of kaolin seemed to be effective in reducing both leaf and grape temperatures and increasing the leaf water potential. This allows grapevines to maintain high photosynthetic activity, preventing irreversible photoinhibition [125]. Both kaolin and zeolite applications enhance the expression of genes related to anthocyanin synthesis, with zeolite being more effective in activating these compared to kaolin. This suggests that these two mineral-based foliar applications may operate though distinct mechanisms [126]. Notably, several studies suggest that the effectiveness of these applications may increase in response to the severity of the environmental stress. However, despite the physiological benefits, their effects on grape quantity and quality are not consistent and appear to be influenced by the environmental conditions, grapevine varieties, and annual climatic variability. There is still a limited number of studies that have investigated the effect of foliar applications under water deficit and heat stress conditions. Therefore, further research is urgently needed to explore how these compounds behave under combined water and heat stress. In particular, using -omics approaches could significantly enhance our understanding of the underlying physiological and molecular mechanisms.

### 3.5. Effect of Foliar Applications on Quality Parameters

Foliar applications of biostimulants, phytohormones, and reflective antitranspirants have emerged as a promising viticultural practice to enhance grapevine resilience under abiotic stress conditions, such as salinity, drought, and heat. In addition to their protective effects, these compounds may also influence the biochemical composition of grapes and wines, particularly in terms of their secondary metabolites.

Among these metabolites, phenolic compounds have received attention due to their associated health benefits and their critical role in the organoleptic properties of grapes and wines [93,100]. These compounds are synthesized though the phenylpropanoid pathway, starting with the amino acid phenylalanine [131]. Phenolic compounds are classified into two major groups: non-flavonoids (i.e., stilbenes, hydroxycinnamic and hydroxybenzoic acids) and flavonoids (i.e., flavonols, flavanols, and anthocyanins) [132,133]. Additionally, aroma compounds contribute to the flavor and quality of grapes and wines [131]. These volatile secondary metabolites are categorized by their origin: primary aromas are derived from the grape; secondary aromas are produced during fermentation; and tertiary aromas develop during wine aging and maturation [134].

Targeted transcriptional analysis using qPCR showed that several genes involved in the biosynthesis of phenolic compounds were differentially expressed in response to various foliar applications. These genes include grapevine *phenylalanine ammonia lyase 1* (*VvPAL1*), *cinnamate-4-hydroxylase 1* (*VvC4H1*), *stilbene synthase 1* (*VvSTS1*), *chalcone synthase 1* (*VvCHS1*), *flavonol synthase 1* (*VvFLS1), dihydroflavonol reductase* (*VvDFR*), and *UDP-glucose:flavonol 3-O-glucosyl transferase* (*VvUFGT*) [16]. Among these, *VvPAL1* catalyzes the initial step in the phenylpropanoid pathway, providing a substrate for the activity of *VvCHS1,* which initiates the flavonoid biosynthesis pathway. This pathway leads to the synthesis of various compounds, including anthocyanins [17]. The enzymes *flavonoid 3′-Hydroxylase* (*VvF3′H*) and *flavonoid 5′-Hydroxylase* (*VvF3′5′H*) catalyze the transformation of flavonoid precursors into anthocyanins. *Dihydroflavonol-4-reductase* (*VvDFR*) and *leucoanthocyanidin dioxygenase* (*VvLDOX,* also referred to as *anthocyanidin synthase*, *VvANS*) are essential enzymes in the anthocyanin biosynthesis pathway [135]. For instance, foliar application of polyols or kaolin resulted in increased accumulation of anthocyanins, stilbenes, and total phenolics in berries. This increase is linked to the upregulation of *VvPAL1*, *VvUFGT1*, and *VviSTS1* [16,96]. The application of kaolin also enhances the concentration of flavonoids by increasing the expression of *VvCHS1* [9,17]. Moreover, abscisic acid (ABA) also influences the expression of *VvPAL1*, *VvDFR*, and *VvANS* [136]. The application of γ-polyglutamic acid increases the anthocyanin content by upregulating the expression of *VvPAL*, *VvCHS*, *VvDFR*, and *VvLDOX* [135]. Similarly, melatonin treatments promote skin pigmentation by increasing transcript levels of *VvPAL*, *VvCHS*, *VvDFR*, *VvLDOX*, *VvF3′H*, and *VvF3′5′H* [137]. Moreover, seaweed extract has been found to increase the synthesis of anthocyanins and other phenolics in berries by upregulating *VvCHS1*, *VvF3′H*, *VvF3′5′H*, and *VvUFGT* [21,122]. Moreover, *A. nodosum* selectively activates *VvF3′H* instead of *VvF3′5′H*, thus favoring the accumulation of di-hydroxy B-ring-substituted flavonoids over their tri-hydroxy counterparts [121,122]. As shown in Table 5, various studies have demonstrated that foliar applications can significantly influence the biosynthesis and accumulation of these secondary metabolites. However, the effects vary depending on the type of compound applied, the environmental conditions, and grapevine variety.

## 4. Conclusions

The increasing frequency and intensity of abiotic stresses such as salinity, drought, heatwaves, and their combinations pose significant challenges to viticulture. These stressors, particularly in the context of climate change, negatively affect grapevine growth, physiology, productivity, metabolism, and the quality of grapes and wines. Earlier studies primarily demonstrated the overall benefits of foliar applications of biostimulants, phytohormones, osmoprotectants, trace elements, and reflective antitranspirants to grapevines’ physiology and productivity. However, recent research over the past decade has provided deeper insights into the stress-specific effectiveness of these compounds under adverse conditions. This review highlights that certain foliar applications show promising effects depending on the type of abiotic stress. Under salinity stress, silicon, abscisic acid, and brassinolide were particularly effective. For drought stress, kaolin, seaweed extracts, methyl jasmonate, melatonin, putrescine, and brassinolide demonstrated notable benefits. In the case of heat stress, positive results were observed with kaolin, seaweed extract, and gibberellic acid. When grapevines were subjected to combined water deficit and heat stress, foliar applications of kaolin, zeolite, and CaCO_3_ appeared to mitigate stress effects. This evidence supports a more targeted approach to foliar applications, offering practical tools for promoting sustainable grape and wine production in the face of climate change. However, further research is still needed to optimize the application protocols (such as dose, timing, and frequency) for each compound under specific types of stress, particularly in field conditions. Moreover, the precise mechanisms of action for many of these compounds under stress conditions remain poorly understood. Utilizing -omics approaches (transcriptomics, metabolomics, and proteomics) could significantly enhance our understanding of how these compounds activate plant responses under abiotic stress. In summary, foliar applications can improve grapevine tolerance to salinity, drought, and heat stress by modulating physiological responses and secondary metabolism, thus supporting yield stability and grape quality.

## Figures and Tables

**Table 1:** Effect of foliar applications on grapevines under salinity–alkalinity stress.

Country	Cultivar	Salinity	Foliar Application	Results	Ref.
Iran (hydroponic culture)	H6 (salt tolerant) GhezelUzum (salt sensitive)	NaCl (0, 50, 100 mM)	Silicon (0, 3 mM Na_2_SiO_3_ at 6-leaf stage and continued for two weeks)	Photosynthesis and gas exchange: ↑ photosynthesis Oxidative stress and antioxidants: in H6 ↑ activity of CAT, and APX, ↓ POD and SOD in leaves, in GhezelUzum ↑ CAT, POD, APX, and SOD in leaves Osmotic adjustment: ↑ proline (in GhezelUzum), ↓ sugar content in leaves and roots Leaf mineral content: ↓ Na, Cl, and Na/K ratio, ↑ K and Si in leaves and roots Morphological traits: ↑ shoot and root dry weights Interaction: Salt-tolerant and salt-sensitive genotypes responded differently under salinity stress	[24]
China (pot experiment)	Cabernet Sauvignon	NaCl (0, 100 mM)	Silicon (0, 2 mM K_2_SiO_3_ 9H_2_O)	Photosynthesis and gas exchange: ↑ photosynthesis, stomatal conductance, and transpiration rate Chlorophyll and pigments: − chlorophyll Osmotic adjustment: ↑ total soluble sugars and starch Mineral content: ↓ Na, ↑ Cl and Si Morphological traits: ↑ stem and leaf dry weights Interaction: Silicon in the absence of salt had negative effects on most parameters	[27]
Iran (pot experiment)	Sultana	NaCl (0, 25, 50, 75, 100 mM)	Abscisic acid (0, 100 μM)	Water relations: ↑ relative water content Chlorophyll and pigments: ↑ carotenoids, chlorophylls a, b Oxidative stress and antioxidants: ↓ MDA and H_2_O_2_, ↑ activity of CAT, GPX, and APX Osmotic adjustment: ↑ proline content and total soluble sugars Cell membrane integrity: ↓ electrolyte leakage, improved membrane stability Morphological traits: ↑ height, leaf number, leaf area, and shoot dry matter Mineral content: ↑ Mg, Ca, K, P, NO_3_^−^, Mn, and Fe, ↓ Cl, Na Secondary metabolism: ↑ total flavonoids and phenolics (leaves)	[29]
Egypt (open-field experiment)	Thompson Seedless	NaCl (0, 1000, 2000, 3000 mg L^−1^)	Brassinolide (0, 1, 2 mg L^−1^)	Water relations: ↑ leaf relative water content Chlorophyll and pigments: ↑ chlorophyll a, chlorophyll b, and carotenoids Oxidative stress and antioxidants: ↓ activity of CAT and POD Osmotic adjustment: ↓ proline content Morphological traits: ↑ survival percentage, plant height, stem thickness, number of leaves, leaf area, shoot and root dry weights Anatomical changes: Mitigation of the harmful effects of salinity on leaf anatomy Reversed ultrastructural modifications to cell organelles Mineral content: ↑ N, P, K, ↓ Na Secondary metabolites: ↓ total phenolic content (leaves) Optimal brassinolide concentration: 2 mg L^−1^	[25]
China (pot experiment)	Kyoho	NaCl: Na_2_SO_4_: NaHCO_3_ (0, 50 mM)	Spermidine (0, 0.5 μΜ)	Water relations: ↑ leaf relative water content Photosynthesis and gas exchange: ↑ photosynthetic rate, transpiration rate, stomatal conductance, WUE Chlorophyll and pigments: ↑ chlorophylls a and b and carotenoids Oxidative stress and antioxidants: ↓ MDA, H_2_O_2_, and O_2_^−^ ↑ activity of CAT, SOD, POD, and APX Osmotic adjustment: ↑ proline content and total soluble sugars Morphological traits: ↑ leaf dry and fresh weights Mineral content: ↑ K, ↓ Na	[32]
China (pot experiment)	Cabernet Sauvignon	NaHCO_3_ (0, 200 mM)	Abscisic acid (50, 100 μM)	Photosynthesis and gas exchange: ↑ photosynthetic rate ↓ intercellular CO_2_ concentration Chlorophyll and pigments: ↑ chlorophyll content Oxidative stress and antioxidants: ↓ MDA, H_2_O_2_, and O_2_^−^, ↑ activity of CAT, SOD, and APX Osmotic adjustment: ↑ proline content Mineral content: ↑ K, ↓ Na Secondary metabolites: ↑ flavonoids	[33]
Turkey (open-field experiment)	Narince	Alkaline soil	*Ascophyllum nodosum* extract (first application of 0.3 g L^−1^ when the shoots were about 20 cm, with 15-day intervals, and a final (fourth) application when the berries were approximately 0.5 mm thick)	Chlorophyll and pigments: ↑ chlorophyll content Morphological traits: − leaf dry and fresh weight and leaf area Quantity traits: − cluster number and weight, berry length and diameter, and yield ↑ berry weight and volume (only one year) Quality traits: − TSS, TA, and pH Mineral content: − N, P, K, Mg, and Cu, ↑ Ca, Zn, S, and B, ↓ Mn, Fe, and Al	[85]

↑: significant increase; ↓: significant decrease; −: no significant effect.

**Table 3:** Effect of foliar applications on grapevines under heat stress.

Country	Cultivar	Temperature	Foliar Application	Results	Ref.
Italy (open-field experiment)	Verdicchio	T_max_ > 35 °C	Kaolin (different dosages depending on year of the experiment)	Photosynthesis and gas exchange: ↓ or ↑ stomatal conductance and transpiration (year depended) Quantity traits: − berry weight Quality traits: ↓ or − TSS, pH, ↑ or − TA	[116]
Italy (open-field experiment)	Sauvignon Blanc	T_max_ > 30 °C	Kaolin particle film (1.5 kg/100 L at veraison and 3 kg/100 L after 10 and 20 days)	Leaf temperature: ↓ (only the second year) Photosynthesis and gas exchange: − stomatal conductance, transpiration, and WUE Chlorophyll and pigments: − chlorophyll content (SPAD) Quality traits: ↓ TSS, ↑ TA, − pH	[117]
Portugal (open-field experiment)	Touriga-Nacional	T_max_ > 35 °C for 36 days	Kaolin particle film (5% at pre-veraison)	Oenological parameters: ↑ TA, tartaric acid, ↓ pH, alcoholic degree Deep reddish color Secondary metabolites: ↑ total anthocyanins and phenolics	[59]
USA (open-field experiment)	Syrah	T_max_ > 40 °C	Kaolin (14, 28, and 56 kg/ha applied to pea-sized berries at intervals of 15 and 26 days)	Leaf temperature: − or ↓ Photosynthesis and gas exchange: − or ↑ stomatal conductance Quantity traits: − yield, clusters/vine, cluster and berry mass, berries/cluster Quality traits: − TSS, pH, and TA (TA ↑ only the third year) Secondary metabolites: − total anthocyanins, tannins, catechin, and quercetin	[118]
China (open-field experiment)	Meili	T_max_ > 35 °C	Kaolin particle film (6% at pre-veraison)	Quantity traits: ↓ 100-berry weight Quality traits: ↑ TSS, ↓ TA Secondary metabolites: ↑ total anthocyanins, phenolics, and flavonoids in skin ↓ or − total flavanols in skin, ↑ or − tannins in skin	[119]
Greece (open-field experiment)	Crimson Seedless	T_max_ > 30 °C Irrigation	*Ecklonia maxima* (3 L ha^−1^, two sprays) *Ascophyllum nodosum* (4 g L^−1^ vine^−1^, five sprays), inactivated wine yeast (1.5 kg ha^−1^, two sprays), ethephon (250 ppm, two sprays), Sunred (4 L ha^−1^, two sprays)	Quantity traits: ↑ yield, cluster and berry weight, berry length and diameter ↓ berry skin mass. − clusters/vine, cluster length and width Quality traits: − TSS (except from Sunred, which ↑), ↓ TA Secondary metabolites: − anthocyanins (except from Sunred, which ↑) Effectiveness: Sunred was the most effective canopy treatment	[120]
Italy, USA (open-field experiment)	Sangiovese (Italy) Pinot Noir Cabernet Franc (USA)	Italy = warmer and dryer conditions than those in the USA	*Ascophyllum nodosum* (1.5 kg/ha, 3 kg/ha, five applications, first three weeks after the formation of pea-size berries, intervals of ten to twenty days)	Italy Quantity traits: − yield, clusters/vine, cluster weight Quality traits: − TSS, pH, and TA Secondary metabolites: ↑ total anthocyanins and phenolics in skin USA (both varieties) Quantity traits: − yield, clusters/vine, berries/cluster, cluster and berry weight Quality traits: − TSS, pH, TA Secondary metabolites: ↑ total anthocyanins in skins, − phenolics in skins	[5]
Brazil (open-field experiment)	Niágara Rosada	T_max_ > 30 °C Irrigation	*Ascophyllum nodosum*, *Hypnea musciformis*, *Lithothamnium* sp., *Sargassum vulgare* extracts (0.6% 20 days after breaking dormancy and at bloom, fruit set, and veraison)	Photosynthesis and gas exchange: ↑ photosynthetic rate, stomatal conductance, WUE, and intercellular CO_2_ concentration Chlorophyll and pigments: ↑ chlorophyll content (only *A. nodosum*) Mineral content: ↑ K, Mg, B, Cu, and Zn in leaves Quantity traits: ↑ yield (only *A. nodosum*) Effectiveness: (AN > LS > HM ≈ SV)	[87]
Italy (open-field experiment)	Sangiovese	T_max_ > 33 °C	*Ascophyllum nodosum* extract (3 g/L one week after full veraison and 15 days after)	Photosynthesis and gas exchange: ↑ net assimilation rate and stomatal conductance Quantity traits: − berry weight Quality traits: ↓ TSS, − pH and TA Secondary metabolites: − individual anthocyanins, hydroxycinnamic acids, quercetin derivatives, and total phenolics	[121]
Italy (open-field experiment)	Sangiovese	T_max_ > 33 °C	*Ascophyllum nodosum* extract (1.5 kg ha^−1^, six applications from 3 weeks after the formation of pea-size berries to harvest)	Quantity traits: − yield, bunches/vine, bunch weight, berries/bunch, and berry weight Quality traits: − TSSs, pH, and TA Secondary metabolites: ↑ total anthocyanins and phenolics	[122]
Romania (open-field experiment)	Moldova	T_max_ > 30 °C	Gibberellic acid (25, 50, and 100 mg/L when 80% of caps had fell) Cropmax (1, 2.5, and 5 mL/L at full inflorescence and fruit set and when berries were pea-sized)	Photosynthesis and gas exchange: ↓ or − photosynthetic rate, − stomatal conductance Chlorophyll and pigments: ↓ chlorophylls a and b − carotenoids Quantity traits: ↑ marketable yield (only GA_3_), − seed and skin weight, seeds/berry Quality traits: − TSS, pH, and TA (GA_3_) ↑ TSS and TA (Cropmax 2.5 mL/L) Secondary metabolites: ↑ total anthocyanins and phenolics (Cropmax) ↓ total anthocyanins and phenolics (GA_3_)	[20]
China (pot experiment)	Thompson Seedless	T_max_ > 40 °C Irrigation	β-Myrcene BaZFP924 protein Aspergone	Photosynthesis and gas exchange: ↑ photosynthetic rate Chlorophyll and pigments: ↑ chlorophyll content (SPAD) Morphological traits: ↑ height and stem thickness − root and internode length Effective order: BaZFP924 protein > β-Myrcene > Aspergone	[63]

↑: significant increase; ↓: significant decrease; −: no significant effect.

**Table 4:** Effect of foliar applications on grapevines under water deficit and heat stress.

Country	Cultivar	Temperature Irrigation	Foliar Application	Results	Ref.
Italy (open-field experiment)	Sangiovese	T_aver_ = 26.2 °C (August) No irrigation	Kaolin and zeolite (3 kg L^−1^ at the beginning and end of veraison)	Leaf temperature: ↓ (only the first year) Berry temperature: ↓ (kaolin) Water relations: − stem water potential Photosynthesis and gas exchange: − stomatal conductance and leaf assimilation rate Quantity traits: − yield, bunch, and berry weight	[10]
Greece (open-field experiment)	Roditis	T_aver_ = 27.9 °C No irrigation	Kaolin and zeolite (3% at the beginning of veraison and one week later)	Leaf temperature: ↓ in the morning and at midday in veraison and harvest (only zeolite) Photosynthesis and gas exchange: ↑ net photosynthesis and WUE in the morning and at midday at veraison and harvest (only zeolite), ↑ stomatal conductance in the morning and at midday at harvest (only zeolite) Quantity traits: ↑ yield, cluster and berry weight, − clusters/vine, and berries/cluster ↓ sunburn necrosis, dehydrated berries, and infected berries Quality traits: − TSS and pH, ↑ TA Secondary metabolites: ↓ or − total phenolics Effectiveness: Zeolite is more effective than kaolin	[6]
Greece (open-field experiment)	Assyrtiko	T_max_ > 30 °C No irrigation and irrigation	Kaolin and CaCO_3_ (5% at a single dose, 150–160 DOY)	Leaf temperature: ↓ at harvest Photosynthesis and gas exchange: ↑ photosynthesis and stomatal conductance Chlorophyll and pigments: ↑ chlorophyll content(SPAD) (only in non-irrigated vines) Quantity traits: ↑ bunch and berry weight (only in non-irrigated vines) − bunch number/vine, berry length and width ↓ sunburn necrosis and infected berries Quality traits: ↓ TSSs, − or ↓ pH and TA Effectiveness: Kaolin was more effective than CaCO_3_ Interaction: Varying effects depending on year, region, training system, and irrigation	[125]
Greece (open-field experiment)	Assyrtiko Mavrotragano	No irrigation	Kaolin and CaCO_3_ (5% at bunch closure and veraison)	Quantity traits: Varying effects on grape length, width, weight − berry length and width − or ↓ 50-berry weight Quality traits: − TA Varying effect on TSSs, glucose, fructose, tartaric acid, malic acid, and pH Secondary metabolites: Varying effect In general, ↑ phenolic compounds, flavanols, flavonoids, flavones, flavonols, and anthocyanins in the skin Interaction: Varying effects depending on variety, region, and training system	[127]
Italy (open-field experiment)	Sangiovese	T_max_ > 30 °C No irrigation	Kaolin, zeolite (3 kg L^−1^ at the beginning and at full veraison)	Quality traits: − TSS and pH ↑ TA (kaolin only one year) Secondary metabolites: ↑ total anthocyanins (zeolite only one year)	[126]
Portugal (open-field experiment)	Touriga Franca Touriga Nacional Tinta Francisca Vinhao Grenache Borraçal Cornifesto	T_max_ > 40 °C Deficit irrigation	Kaolin (5% at pre-veraison)	Quantity traits: ↑ berry size, ↓ skin/pulp Secondary metabolites: Variable effect − total anthocyanins in whole fruit, ↑ or ↓ total anthocyanins of skin −, ↑, or ↓ total phenolic content and total flavonoids in the berries, skin, and seeds Interaction: Varying effect depending on variety	[7]
Portugal (open-field experiment)	Touriga Franca Touriga Nacional	T_max_ > 40 °C Deficit irrigation	Kaolin (5% at pre-veraison)	Leaf temperature: − or ↓ temperature Chlorophyll and pigments: − or ↑ chlorophyll content Osmotic adjustment: ↑ or ↓ proline Interaction: Varying effect depending on variety, region, year, and phenological stage	[128]
Portugal (open-field experiment)	Touriga Franca Touriga Nacional	T_max_ > 40 °C Deficit irrigation	Kaolin (5% at pre-veraison)	Hormonal balance: −, ↑, or ↓ ABA, IAA, and SA Quality traits: −, ↑, or ↓ soluble sugars, − TA and pH Secondary metabolites: − or ↓ total phenols and flavonoids; −, ↑, or ↓ total anthocyanins; ↑ tannins Interaction: Varying effect depending on variety, year, and phenological stage	[105]
Portugal (open-field experiment)	Touriga Franca Touriga Nacional	T_max_ > 40 °C Deficit irrigation	Kaolin (5% at pre-veraison)	Hormonal balance: ↑ or ↓ SA and ABA Quality traits: ↓ or − soluble sugars and − TA, pH, malic acid, and tartaric acid Secondary metabolites: ↑ or − total phenols, flavonoids, and total anthocyanins and − tannins Interaction: Varying effect depending on variety, year, and phenological stage	[129]
Spain (open-field experiment)	Verdejo	T_max_ > 35 °C Deficit irrigation	Kaolin (5%, three times between fruit set and veraison)	Leaf temperature: ↓ Photosynthesis and gas exchange: ↑ maximum quantum efficiency of photosystem II, photochemical quenching, and electron transport rate, ↓ basal fluorescence Quality traits: − TSSs and TA, ↓ pH and total phenolics	[130]
Italy (pot experiment)	Sangiovese	T_max_ > 30 °C Deficit irrigation	Zeolite (3% at the beginning and end of veraison)	Leaf temperature: ↓ Photosynthesis and gas exchange: ↑ net photosynthesis and stomatal conductance Quantity traits: − yield and cluster and berry weight Quality traits: − TSSs, pH, and TA Secondary metabolites: ↑ total anthocyanins	[124]
Italy (open field experiment)	Barbera	T_max_ > 35 °C No irrigation	Proline-rich specific yeast derivative	Leaf and cluster temperature: − Water relations: ↑ leaf water potential Photosynthesis and gas exchange: ↑ stomatal conductance and assimilation rates, − WUE Chlorophyll and pigments: ↑ β-carotene and chlorophylls a, b Oxidative stress and antioxidant defense: ↓ H_2_O_2_ Osmotic adjustment: ↑ proline Quantity traits: ↓ cluster sunburn − clusters/vine and skin weight ↑ yield, cluster weight, cluster compactness, and berry weight Quality traits: ↓ TSS and TSS/TA − pH, TA, tartaric, and malic acid Secondary metabolites: − anthocyanins and phenolics (mg/g); ↑ anthocyanins and phenolics (mg/berry)	[63]

↑: significant increase; ↓: significant decrease; −: no significant effect.

**Table 5:** Effect of foliar applications on grape and wine quality parameters.

Country	Cultivar	Foliar Application	Results	Ref.
Italy	Pinot Noir	Chitosan	Secondary metabolites: ↓ citronellol, 2,4-di-tert-butylphenol ↑ non-volatile phenolics Wines sensorially described as having “unpleasant flavors” and “odors”	[91]
Italy	Sangiovese Cabernet Sauvignon	Chitosan	Secondary metabolites: ↑ catechin, epicatechin, and procyanidin B2 in Cabernet Sauvignon − anthocyanins and flavonols or t-resveratrol in berry skin	[92]
Italy	Chardonnay, Cortese, Nebbiolo	Inactivated dry yeast	Quality traits: − reducing sugars, pH, and TA Secondary metabolites: Varying effect depending on variety and year	[138]
Italy	Negro Amaro, Primitivo	Inactivated dry yeast	Quality traits: − reducing sugars, pH, and TA Secondary metabolites: ↑ aroma potential (↑ carotenoids − C13-norisoprenoid precursors)	[139]
Italy	Scarlotta Seedless Crimson Seedless Red Globe	Inactivated dry yeast	Secondary metabolites: ↑ anthocyanins (particularly in Crimson Seedless, which is characterized by pigment concentrations lower than those in Scarlotta Seedless and Red Globe)	[140]
Italy	Sauvignon Blanc	Inactivated dry yeast	Quality traits: − sugars and acidity Secondary metabolites: ↑ aroma precursors	[69]
USA	Chambourcin	Inactivated dry yeast	Quality traits: ↓ pH, − TSSs Secondary metabolites: ↑ malvidin-, delphinidin-, petunidin-3-O-glucoside, and total anthocyanins	[95]
China	Ruidu Hongyu	2,4-epibrassinolide Jasmonic acid	Secondary metabolites: ↑ anthocyanins, phenolics, and flavonoids	[141]
Spain	Tempranillo	Methyl jasmonate Chitosan Yeast extract	Secondary metabolites: ↓ terpenoids, C13 norisoprenoids, benzenoids, and esters (except for yeast extract)	[132]
Spain	Tempranillo	Methyl jasmonate, chitosan, yeast extract	Quality traits: − TSSs, TA, and pH in berries, ↑ TA in wine Secondary metabolites: − total anthocyanins, flavonols, flavanols, and phenolic acids in grapes and wines ↑ trans-resveratrol in grapes ↑ anthocyanins in wine for methyl jasmonate Varying effect on individual anthocyanins	[93]
Spain	Tempranillo	*Ascophyllum nodosum* extract	Secondary metabolites: ↑ total stilbenes, malvidin-3-glc, myricetin-3-glc, and myricetin-3-gal in grapes Varying effect on phenolic compounds depending on year	[133]
Spain	Tempranillo	*Ascophyllum nodosum* extract	Secondary metabolites: ↑ several individual terpenoids, C13 norisoprenoids, esters, benzenoids, alcohols, carbonyl compounds, and C6 compounds in must and wine	[142,143]
Greece	Merlot	*Ascophyllum nodosum* extract	Quality traits: − TSS, TA, malic acid, and tartaric acid Secondary metabolites: − total anthocyanins in berries, − or ↓ total phenolic index, ↑ seed tannin index	[144]
Turkey	Tarsus Beyazı	*Ascophyllum nodosum* extract	Quality traits: ↓ TSS and pH, ↑ TA	[145]
Italy	Corvina	Animal- and plant-derived protein hydrolysates (casein, soybean, and lupin)	Quality traits: ↑ TSS, ↓ TA and malic acid Secondary metabolites: ↑ total anthocyanins	[83]
Italy	Merlot	Protein hydrolysates of plant origin (Trainer, Stimtide)	Quality traits: ↓ TSSs, ↑ TA, − pH, tartaric acid, and malic acid Secondary metabolites: − total anthocyanins and phenolics	[146]
Romania	Feteasca Regala Riesling Italian	Humic acid	Quality traits: ↑ TSS, ↓ TA	[77]
China	Cabernet Sauvignon Riesling	Fulvic acid	Secondary metabolites: ↑ total phenols, flavonoids (Riesling grapes), total tannin, individual flavanols, and volatiles (Cabernet Sauvignon grapes and wine) ↓ individual phenolic acids and flavonols (Cabernet Sauvignon wine)	[147]
Italy	Ribolla Gialla	Biostimulant made using Fabaceae tissue rich in triacontanol	Quality traits: ↑ TSS, citric acid, − pH, TA, tartaric acid, and malic acid ↓ lactic acid	[79]
Italy	Sangiovese	Methyl jasmonate	Secondary metabolites: ↑ aroma compounds, ↑ several monoterpenes, norisoprenoids, and esters	[134]
Spain	Tempranillo	Methyl jasmonate	Quality traits: ↑ TSSs, amino acids; − pH, TA	[148]
Spain	Garnacha	Methyl jasmonate	Quality traits: − TSS, pH, TA, tartaric acid, and malic acid Secondary metabolites: ↑ total anthocyanins, flavonols, hydroxybenzoic acids, and several individual compounds − total phenolic content, flavanols, and antioxidant capacity	[100]
Spain	Tempranillo Graciano	Methyl jasmonate	Quality traits: − TSS, pH, TA, tartaric acid, and malic acid Secondary metabolites: − flavonols, flavanols, and hydroxybenzoic acids ↑ some anthocyanins and stilbenes	[131]
Spain	Tempranillo	Methyl jasmonate	Quality traits: − TSS, pH, TA, tartaric acid, malic acid, fructose, and glucose Secondary metabolites: ↓ total phenolic content − terpenoids and C13 norisoprenoids	[149]
Egypt	Crimson Seedless	Abscisic acid Methyl jasmonate Ethephon melatonin	Quality traits: ↑ berry firmness, TSS, ↓ TA Secondary metabolites: ↑ total anthocyanins, phenolics, and antioxidant capacity	[150]
China	Muscat Hamburg	Abscisic acid	Quality traits: ↑ TSS, glucose, and fructose; ↓ TA, tartaric, malic, and citric acids Secondary metabolites: ↑ total and individual anthocyanins	[151]
China	Merlot	Abscisic acid	Quality traits: ↑ total sugar, − TA Secondary metabolites: ↑ total phenolics, total and Individual anthocyanins	[152]
Chile	Carmenere	Abscisic acid	Quality traits: − TSS, TA Secondary metabolites: ↓ non-methylated anthocyanins (delphinidin and petunidin), flavonols ↑ methylated anthocyanins (malvidin and peonidin), tannins	[136]
China	Ruiduhongyu	Abscisic acid	Quality traits: ↑ TSS, ↓ TA Secondary metabolites: ↑ polyphenols, flavonoids, resveratrol	[153]
Italy	Crimson Seedless	Abscisic acid Harpin proteins	Quality traits: − TSS, pH, TA, and firmness Secondary metabolites: ↑ individual anthocyanins (especially abscisic acid)	[154]
China	Red Globe	Abscisic acid, Sunred	Quality traits: ↑ TSS, ↓ TA Secondary metabolites: ↑ total anthocyanins	[155]
China	Summer Black	Melatonin	Quality traits: ↑ TSS, fructose, glucose, and sucrose; ↓ TA Secondary metabolites: ↑ or ↓ total and individual anthocyanins	[137]
Argentina	Malbec	Abscisic acid Gibberellic acid	Secondary metabolites: ↑ mono- and sesquiterpenes in berries, ↑ (ABA) and ↓ (GA3) anthocyanins	[156]
China	Marselan	γ-polyglutamic acid, alginic acid	Secondary metabolites: ↑ total phenolics and antioxidant capacity in grapes and wine	[135]
China	Crimson Seedless Red Barbara Summer Black Hutai No. 8	Selenium	Quality traits: ↑ TSS, soluble protein, and vitamin C ↓ organic acids Secondary metabolites: ↑ procyanidins − resveratrol	[157]
Spain	Monastrell	Oak extract	Quality traits: ↓ TSS in grapes, ↑ TA in grapes, ↓ in wine, − pH, tartaric acid, and malic acid Secondary metabolites: ↑ total anthocyanins and phenols in wine ↑ gallic acid, hydroxycynnamoyltartaric acids, acylated anthocyanins, flavanols, and stilbenes	[158]
Italy	Sangiovese	Antitranspirant di-1-p-menthene	Quality traits: ↓ TSS, − pH, TA, tartaric acid, and malic acid	[159]
Egypt	Thompson Seedless	Vitamin B Glutamic acid *Amphora ovalis* extract	Quality traits: ↑ TSS, ↓ TA	[160]

↑: significant increase; ↓: significant decrease; −: no significant effect.
